# Does increased provider effort improve quality of care? Evidence from a standardised patient study on correct and unnecessary treatment

**DOI:** 10.1186/s12913-023-09149-5

**Published:** 2023-02-23

**Authors:** Jessica Julia Carne King, Timothy Powell-Jackson, James Hargreaves, Christina Makungu, Catherine Goodman

**Affiliations:** 1 London School of Hygiene and Tropical Medicine, 15-17 Tavistock Place, WC1E 9SH London, UK; 2 Ifakara Health Instiute, Plot 463, Kiko Avenue, P.O. Box 78 373, Mikocheni, Dar es Salaam, Tanzania

**Keywords:** Quality of care, Overprovision, Provider effort

## Abstract

**Background:**

Poor quality of care, including overprovision (unnecessary care) is a global health concern. Greater provider effort has been shown to increase the likelihood of correct treatment, but its relationship with overprovision is less clear. Providers who make more effort may give more treatment overall, both correct and unnecessary, or may have lower rates of overprovision; we test which is true in the Tanzanian private health sector.

**Methods:**

Standardised patients visited 227 private-for-profit and faith-based facilities in Tanzania, presenting with symptoms of asthma and TB. They recorded history questions asked and physical examinations carried out by the provider, as well as laboratory tests ordered, treatments prescribed, and fees paid. A measure of provider effort was constructed on the basis of a checklist of recommended history taking questions and physical exams.

**Results:**

15% of SPs received the correct care for their condition and 74% received unnecessary care. Increased provider effort was associated with increased likelihood of correct care, and decreased likelihood of giving unnecessary care. Providers who made more effort charged higher fees, through the mechanism of higher consultation fees, rather than increased fees for lab tests and drugs.

**Conclusion:**

Providers who made more effort were more likely to treat patients correctly. A novel finding of this study is that they were also less likely to provide unnecessary care, suggesting it is not simply a case of some providers doing “more of everything”, but that those who do more in the consultation give more targeted care.

**Supplementary Information:**

The online version contains supplementary material available at 10.1186/s12913-023-09149-5.

## Background

Expanding good quality healthcare, accessible to all, is a key part of the universal health coverage agenda [1, 2]. However, quality of care has been shown to be severely lacking in many settings, including in low- and middle-income countries (LMICs). There is widespread evidence of providers making incorrect diagnoses of serious illnesses [3, 4], not carrying out the correct clinical procedures [5, 6], and failing to prescribe the correct medications [7–9]. Poor quality of care has been estimated to be responsible for 10–15% of all deaths in LMICs[10].

Poor quality of care around the world reflects both underprovision, the failure to use appropriate and effective medical interventions, and overprovision, defined as medical services that are more likely to cause harm than good [11, 12]. Patients can receive overprovision in addition to the correct care, or alongside underprovision [13]. While overprovision is often framed as a concern in high income countries [14, 15], it can be overlooked when examining quality of care in LMICs, where underprovision is widespread [12]. However, recent studies have found substantial evidence of unnecessary tests and medications in LMIC settings [3, 6, 7, 16–19]. Tackling overprovision should be a priority for health systems, as it is wasteful for the system and the individual patient [20], and can cause harm to both patients [21] and public health [22].

In this paper, we study the relationship between provider effort – as reflected by the number and type of actions the provider takes in a consultation, such as asking questions about symptoms and carrying out physical exams, in order to come to a diagnosis and decide on management [23] – and quality of care, as measured by whether the correct management is given. While provider effort could be conceptualised as a component of good quality care in itself, we treat it here as on the pathway to providing correct management [24, 25]. Effort is likely to be a function of multiple factors: workload, intrinsic motivation, clinical knowledge, and training in how to make a diagnosis. At first glance, the relationship between effort and correct management may seem obvious: health care providers who exert greater effort in applying their knowledge can be expected to deliver better quality care. However, this relationship can be complicated by the fact that the health care provider has better information on what care the patient needs than the patient herself, and the patient cannot ascertain the quality of care given even after receiving it. It is possible that better skilled and more motivated health care providers may provide more clinically unnecessary care, because they have more of an opportunity to exploit the informational asymmetry. This may be particularly the case in the private sector, where financial incentives to maximise profits may undermine intrinsic motivation to provide good quality care.

Exploring these relationships is challenging because it is difficult to establish whether the care received by a patient is correct or unnecessary, and because measuring effort in a consultation is not straightforward. In recent years, standardised patients (SPs), who are fieldworkers trained to visit health facilities and act as real patients, have been used to measure quality of care in terms of both the effort exerted by the provider and whether correct management was provided [26]. Such studies have generally found that consultations in which providers exert more effort through longer consultations, asking more questions and doing more physical exams are more likely to result in the SP receiving correct management [16, 17, 27, 28]. However, there is limited evidence on whether those providers who make more effort are more or less likely to provide unnecessary care. Understanding the relationship between effort and the quality of care, and particularly unnecessary care, is key when it comes to choosing the type of policies and interventions needed to improve quality of care: do we need to improve providers’ knowledge, motivate them to exert more effort in a consultation, or change incentive structures to discourage the provision of unnecessary care?

A further complication in understanding the incentives for exerting effort and providing quality care are the different ways that private sector facilities charge patients for their care (even without considering patients who are covered by public or private insurance, which adds further variation). The most common model is to charge a relatively low registration or consultation fee when the patient registers to see a clinician, which is a small proportion of the overall cost after individual tests and drugs are charged for. Some facilities charge a substantial fee for the consultation, perhaps signalling that the clinician’s time and expertise is the main value of the visit to the facility. Others do not charge a consultation fee at all, either because the facility is not-for-profit and only drugs are paid for, or because the clinician’s time is seen as ‘included’ in the final bill for drugs and tests.

In this paper, we examine the relationship between provider effort and both correct care and unnecessary care in private health facilities in Tanzania, in order to explore the extent to which under and over treatment are associated with provider effort. We further explore the relationship between provider effort and fees charged for services to understand the reward mechanisms for delivering good quality care.

## Methods

### Study setting and participants

Data was collected in May-June 2018 as part of the endline survey of a randomised controlled trial of the SafeCare quality improvement programme, described elsewhere [29]. 228 private-for-profit and not-for-profit health facilities participated, located in rural and urban areas across 18 regions of mainland Tanzania. The sample included dispensaries (the lowest level of health facility, often staffed by a single clinical officer with three years of post-secondary clinical training), health centres (a larger facility with more staff and which may admit patients) and hospitals (which all have inpatient wards and usually have a fully qualified doctor on staff).

The Tanzanian private medical sector plays an important role in the provision of care; analysis of national health accounts estimates that 30.1% of health expenditure in medical facilities is in the private sector (19.9% in non-for-profit facilities, 11.2% in for-profit facilities) [30]. Data extracted from the Health Facility Registry in 2022 suggest that there are around 3000 private health facilities in the country, 83% of which are dispensaries and clinics, 10% health centres and 7% hospitals[31].

### Standardised patient data collection

Standardised patients (SPs) are healthy fieldworkers, trained to present at health facilities acting as real patients, and report a standardised set of symptoms and history to the clinician. Written consent for SP visits was sought from the facility manager, one to four months before the SP visits, without giving details of the presenting conditions of SPs. Two SPs visited each of the health facilities, one presenting a case of asthma and the other a case of suspected TB. During the consultation, they made an initial statement of their presenting complaint, shown in Table 1. Further details of other symptoms and history (also shown in Table 1) were only given if the clinician asked a relevant question.

Immediately after finishing a visit to a facility, SPs completed a debriefing questionnaire using ODK Collect on mobile phones, reporting on history taking and physical exams carried out by the clinician, laboratory tests ordered and their results, diagnosis given by the doctor, treatments prescribed and dispensed, and any fees paid. SPs underwent laboratory tests including fingerprick tests for malaria and provided urine samples if requested by the clinician, but refused venous blood draws, sputum tests, X-rays and HIV tests (still recording them as ordered). They bought any drugs prescribed but avoided any treatments which would be administered at the facility, such as injections or drips. SPs paid for all services in cash. A supervisor verified forms at the end of each day, and collected and labelled any drugs bought. Drugs were checked against the form by the study team at the end of fieldwork. A follow-up telephone survey with facility managers assessed whether providers detected any SPs.

### Measuring provider effort

Provider effort was measured from a checklist of history taking and physical examinations. There were 33 checklist items for the asthma case and 29 for the TB case. The checklist was developed using Tanzanian Standard Treatment Guidelines [32] and in consultation with a panel of expert pharmacists and clinicians. Item response theory (IRT), the details of which are given in the appendix, was used to construct a continuous measure of effort for each case [33]. IRT allows each checklist item to vary in its difficulty and ability to discriminate between providers, to create a measure which more accurately captures the amount of effort exerted in the consultation than simply the proportion of checklist items completed.

### Outcomes

Details of the correct management of SPs are given in Table 1. Required drugs and lab tests come from the Tanzanian Standard Treatment Guidelines [32]. Details of the classification of drugs and tests as palliative, appropriate and unnecessary have been published elsewhere [7]. Quality of care is measured with two binary outcomes: correct management (SP was prescribed or ordered the required drugs and tests) and unnecessary care (SP was prescribed or ordered any test not categorised as appropriate or any drug not categorised as required or palliative). Correct management and unnecessary care are not mutually exclusive, and can occur within the same SP visit. The unnecessary care outcome is equivalent to overprovision.

Total fees for all services received were converted from Tanzanian shilling to US dollars using the World Bank official exchange rate average for 2018 (2,263.78 TZS = 1.00 USD). Where available, a breakdown of separate fees paid for consultation with the clinician, lab tests and drugs is reported.

**Table 1:** Standardised patient (SP) case presentation and correct management

Case	Initial presentation	Further details given if probed	Required drugs and tests	Palliative drugs¹	Appropriate tests²
Asthma	“I have had a problem with breathing, and last night it became terrible”	Shortness of breath when moving furniture/cleaning. Wheezing and non-productive cough throughout attack. Attacks at night for a year with increasing frequency and severity. Attacks brought on by cleaning or physical activity. Had coughing fits as a child, and a sibling with a similar problem.	Prescription of salbutamol or other beta-2 antagonist or steroid inhaler.	Other${ \beta }_{2}$antagonists and steroids, antihistamines, xanthines.	Allergy tests, ECG, HIV, X-ray.
TB	“I have had a cough that is not getting better”	Productive cough for three weeks, one week course of amoxicillin without improvement. Low grade fevers, chest pain, loss of appetite, weight loss, night sweats.	Order or refer for sputum TB testing (including referral to a higher-level public health facility which could test for TB, even if testing was not mentioned).	Cold and flu combinations, cough syrups, NSAIDs and paracetamol.	Complete blood count, HIV, malaria, X-ray, Widal.

¹Drugs which are suitable for managing symptoms associated with the condition, and therefore not classified as unnecessary

²Tests which may give the provider useful information in planning management of the patient

### Analytical approach

We used multivariate linear regression to identify factors associated with provider effort. We adjusted for SP fixed effects, SP case type and intervention arm, and included the following factors of interest: gender of provider, the proportion of outpatient clinicians who were doctors with medical degrees (as opposed to a lower cadre such as clinical officer), whether outpatient clinicians in the facility were paid a fixed salary only or some sort of bonus or other incentive, level (hospital, health centre, or dispensary), location (urban, peri-urban, or rural), whether the facility was for-profit or not-for-profit, and whether the facility had any revenue from private or public insurance funds.

We used modified Poisson regression models to estimate the relationship between provider effort and quality of care. We used two separate models for two quality of care outcomes: correct management, and unnecessary care. We then took three approaches to modelling: Model (1), the base model, included effort, SP fixed effects, SP case type (asthma or TB) and SafeCare invention arm. This was to estimate the effect of effort without adjustment. Model (2) additionally included characteristics related to the provider, their skill level and their incentives. These variables were gender of provider, provider payment mechanism and provider qualifications. Finally, model (3) added wider characteristics of the facility: level, location, profit status and insurance revenue. We conducted a sub-group analyses on for-profit and non-for-profit facilities separately. We also conducted an analysis of the intensive margins of unnecessary care using Poisson regression models and two outcomes: count of unnecessary drugs and count of unnecessary tests, with the same modelling approach. We conducted sensitivity analyses comparing quality of care outcomes in SPs who were referred to other facilities or asked to return for follow up, and those who were not. These are presented in the appendix.

The relationship between effort and total fees paid was estimated using linear regression models, with three modelling approaches as described above. To further understand the determinants of fees paid, separate models were used to estimate consultation fees, lab fees and drugs fees. As a sensitivity analysis, we also modelled fees including the quality of care outcomes as independent variables, and summarised outcomes with consultation fees dichotomised into high (> 1USD) and low (< 1USD). These are presented in the appendix.

## Results

All 228 facilities which were open at the time of seeking consent agreed to visits from SPs. Of these, one facility was only open to staff who worked at a private organisation and so SPs could not be sent. All 227 remaining facilities received a visit from an SP presenting the asthma case and an SP presenting the TB case. Characteristics of facilities and providers are described in Table 2.

**Table 2:** Facility and provider characteristics

*Facility characteristics (n = 227)*	%
Level	
Dispensary	55.1
Health centre	30.0
Hospital	15.0
Ownership type	
Private-for-profit	43.6
Private not-for-profit	56.4
Urbanisation	
Urban	30.8
Peri urban	26.9
Rural	42.3
Payment of outpatient clinicians	
Fixed salary only	81.1
Bonus or payment based on volume or revenue	18.9
Proportion of doctors/medical officers among three highest qualified outpatient staff	
0/3	80.7
1/3	16.3
2/3	2.2
3/3	0.9
Exposure to insurance	
Has insurance income	65.2
No insurance income	34.8
*Provider characteristics (n = 454)*	
Male	76.0
Female	24.0

15% of SPs received the correct management for their condition; this was 25% among the TB cases and 6% for asthma (Table 3). 74% of all SPs received some unnecessary care: 86% of TB SPs and 62% of asthma SPs. The mean fee paid by TB SPs was USD 4.97, compared to USD 3.76 by asthma SPs. This difference seems to be almost entirely due to higher costs for drugs paid by TB SPs (USD 3.40 vs. USD 2.10). An average of 10.5 recommended history taking questions and physical exams were done in each consultation, around one third of total recommended actions (there were 29 recommended actions for TB and 33 for asthma).

A total of 534 unnecessary drugs were prescribed to the SPs. The majority of unnecessary drugs (n = 301) were antibiotics, the most common type of which were beta-lactam antibacterials and penicillins (n = 191), followed by macrolides, lincosamides and streptogramins (n = 42). Other frequently prescribed unnecessary drugs were antihistamines (n = 44, only unnecessary for TB), analgesics (n = 42, only unnecessary for asthma) and steroids (n = 28, only unnecessary for TB). 147 unnecessary tests were recommended to the SPs. The tests which were most frequently recommended unnecessarily were urinalysis (n = 32), malaria microscopy or rapid diagnostic test (n = 29, only unnecessary for asthma) and stool examination for intestinal worms (n = 21). Full lists of unnecessary drugs and tests are given in Appendix tables A7, A8 & A9.

**Table 3:** Consultation outcomes, effort, and fees paid

	Asthma mean (sd)	TB mean (sd)	All mean (sd)
**Outcome of consultation**			
Correct management (n = 454)	0.06 (0.23)	0.25 (0.43)	0.15 (0.36)
Unnecessary care (n = 454)	0.62 (0.49)	0.86 (0.34)	0.74 (0.44)
**Provider effort**			
Number of checklist items carried out (n = 453)¹	10.91 (4.23)	10.13 (4.07)	10.52 (4.16)
Proportion of checklist terms carried out (n = 453)	0.33 (0.13)	0.35 (0.14)	0.34 (0.13)
**Fees paid**			
Total fee USD (n = 453)	3.76 (3.14)	4.97 (3.56)	4.36 (3.40)
*Consultation fee (n = 427)*	1.31 (1.89)	1.30 (1.64)	1.30 (1.78)
*Diagnostic tests fees (n = 448)*	0.29 (0.89)	0.31 (0.76)	0.30 (0.83)
*Medicines fees (n = 427)*	2.10 (2.20)	3.40 (2.90)	2.72 (2.63)

^1^Target number of checklist items was 33 for asthma and 29 for TB

Consultations at facilities with at least three doctors on the outpatient staff had an effort score 0.72 standard deviations higher than those without any doctors (p = 0.007, Table 4). Consultations at hospitals had an effort score 0.39 standard deviations higher than those at dispensaries (p=0.008), and those at health centres were 0.23 standard deviations higher than those at dispensaries (p = 0.046).

**Table 4:** Factors associated with provider effort

*Factor*	*Effort IRT score (standard deviations)*
Female provider	0.14 (-0.06–0.34) p = 0.176
Bonus (vs fixed salary)	0.03 (-0.20–0.26), p = 0.817
% of 3 most qualified clinicians who are doctors	0.72 (0.20–1.23), p = 0.007
Hospital (vs dispensary)	0.39 (0.01– 0.67), p = 0.008
Health centre (vs dispensary)	0.23 (0.00–0.45), p = 0.046
For-profit (vs not-for-profit)	-0.00 (-0.24–0.23), p = 0.992
Peri-urban (vs rural)	-0.06 (-0.30–0.17), p = 0.589
Urban (vs rural)	0.16 (0.08–0.40), p = 0.184
Any insurance revenue	-0.02 (0.84– 2.86), p = 0.851

Coefficients are from a multivariate linear regression model adjusting for SP fixed effects, SP case and SafeCare intervention arm as well as all factors listed

Increased effort was associated with correct care (Table 5), with a one standard deviation increase in IRT score associated with a near doubling in relative risk (RR) of receiving correct management (RR = 1.81, p < 0.001), and a reduction in the risk of providing unnecessary care by 8% (RR = 0.92, p = 0.002). The magnitude and direction of these relationships remained similar in models (2) and (3), when adjusting for provider and facility characteristics.

**Table 5:** Effort and quality outcomes

	Correct management	Any unnecessary care
	Relative risk	Relative risk
Base model		
IRT effort	1.81 (1.43–2.30), p < 0.001	0.92 (0.87– 0.97), p = 0.002
Base model + provider characteristics		
IRT effort	1.80 (1.42–2.30), p < 0.001	0.92 (0.87–0.97), p = 0.003
Female provider	1.74 (1.16–2.62), p = 0.007	0.91 (0.80–1.04), p = 0.166
Bonus (vs fixed salary)	1.41 (0.85–2.33), p = 0.182	1.14 (1.01–1.29), p = 0.030
% of 3 most qualified clinicians who are doctors	1.61 (0.54–4.86), p = 0.394	0.86 (0.63–1.17), p = 0.332
Base model + provider characteristics + facility characteristics		
IRT effort	1.87 (1.47–2.38), p < 0.001	0.93 (0.88–0.98), p = 0.009
Female provider	1.58 (1.06–2.36), p = 0.026	0.90 (0.79–1.03), p = 0.138
Bonus (vs fixed salary)	1.58 (0.94–2.67), p = 0.083	1.14 (1.00–1.30), p = 0.059
% of 3 most qualified clinicians who are doctors	1.39 (0.48–4.03), p = 0.548	0.83 (0.60–1.14) p = 0.247
Hospital (vs dispensary)	1.35 (0.75–2.43), p = 0.314	0.91 (0.75–1.10), p = 0.325
Health centre (vs dispensary)	1.26 (0.78–2.05), p = 0.348	1.03 (0.90–1.17), p = 0.704
For-profit (vs not-for-profit)	0.52 (0.29–0.94), p = 0.029	1.03 (0.90–1.19), p = 0.649
Peri-urban (vs rural)	1.86 (1.12–3.10), p = 0.017	1.08 (0.93–1.26), p = 0.290
Urban (vs rural)	1.09 (0.62–1.91), p = 0.761	0.99 (0.85–1.16), p = 0.944
Any insurance revenue	1.55 (0.84–2.86), p = 0.159	1.00 (0.88–1.14), p = 0.991

Relative risks are from modified Poisson regression models. Base model includes adjustment for SP fixed effects, SP case and SafeCare intervention arm

**Table 6:** Intensity of unnecessary care

	Unnecessary drugs	Unnecessary tests
	Relative risk	Relative risk
Base model		
IRT effort	0.89 (0.81– 0.96), p = 0.005	1.09 (0.84– 1.42), p = 0.513
Base model + provider characteristics		
IRT effort	0.90 (0.82–0.98), p = 0.013	1.00 (0.78–1.28), p = 0.9935
Female provider	0.86 (0.70–1.04), p = 0.121	0.84 (0.51–1.39), p = 0.500
Bonus (vs fixed salary)	1.26 (1.04–1.52), p = 0.016	2.00 (1.21–3.31), p = 0.007
% of 3 most qualified clinicians who are doctors	0.57 (0.34–0.96), p = 0.035	3.11 (0.86–11.2), p = 0.083
Base model + provider characteristics + facility characteristics		
IRT effort	0.91 (0.83–0.99), p = 0.030	1.00 (0.78–1.28), p = 0.993
Female provider	0.86 (0.70–1.05), p = 0.132	0.80 (0.48–1.32), p = 0.379
Bonus (vs fixed salary)	1.27 (1.03–1.56), p = 0.026	2.22 (1.33–3.69), p = 0.002
% of 3 most qualified clinicians who are doctors	0.57 (0.33–0.98), p = 0.043	2.69 (0.78–9.24) p = 0.116
Hospital (vs dispensary)	0.98 (0.75–1.28), p = 0.866	1.11 (0.58–2.14), p = 0.748
Health centre (vs dispensary)	1.09 (0.89–1.33), p = 0.401	1.70 (1.04–2.76), p = 0.034
For-profit (vs not-for-profit)	1.18 (0.95–1.46), p = 0.133	0.75 (0.41–1.35), p = 0.331
Peri-urban (vs rural)	1.08 (0.87–1.35), p = 0.482	1.26 (0.71–2.23), p = 0.432
Urban (vs rural)	0.97 (0.77–1.21), p = 0.777	0.92 (0.51–1.65), p = 0.779
Any insurance revenue	0.87 (0.72–1.06), p = 0.169	0.86 (0.50–1.48), p = 0.578

Relative risks are from Poisson regression models with the count of unnecessary items as outcome. Base model includes adjustment for SP fixed effects, SP case and SafeCare intervention arm

Female clinicians were over 50% more likely to correctly manage SPs than male clinicians (RR = 1.58, p = 0.026), despite not exerting any more effort in the consultation. However, provider gender had no statistically significant impact on the likelihood of unnecessary care. There was some evidence that SPs visiting facilities where outpatient providers were paid a bonus or share of revenue were more likely to receive correct management (RR = 1.58, p = 0.083) and unnecessary care (RR = 1.14, p = 0.059) than at facilities which paid a fixed salary.

Compared to not-for-profit facilities, for-profit facilities were about half as likely to provide correct care (RR = 0.52, p = 0.029), but there was no relationship between profit status and providing unnecessary care. Peri-urban facilities were nearly twice as likely as rural ones to correctly manage SPs (RR = 1.86, p = 0.017), but the same increase was not observed in urban facilities, and there was no relationship with unnecessary care. A sub-group analysis examining the relationship between effort and the quality of care outcomes in not-for-profit and for-profit facilities separately suggests there is little heterogeneity (Appendix Table A10).

Analysis of the intensive margin of unnecessary care (Table 6) revealed that while effort was associated with a reduction in number of unnecessary drugs of a similar magnitude to the reduction in any unnecessary care (RR = 0.91, p = 0.03), there was no reduction in unnecessary tests associated with increased effort (RR = 1.00, p = 0.993). Paying a bonus instead of fixed salary was associated with an increase in both number of unnecessary drugs (RR = 1.27, p = 0.26) and tests (RR = 2.22, p = 0.002), a stronger relationship than observed for the overall unnecessary care outcome. An increased number of fully qualified doctors on staff was associated with a decreased number of unnecessary drugs (RR = 0.57, p = 0.043).

**Table 7:** Fees models

	Total fee (n = 452)	Consultation fee (n = 426)	Labs fee (n = 447)	Drugs fee (n = 426)
Base model				
IRT effort	0.51 (0.18–0.55), p = 0.003	0.37 (0.18–0.55), p < 0.001	0.06 (-0.03–0.14), p = 0.180	0.08 (-0.19–0.34), p = 0.563
Base model + provider characteristics				
IRT effort	0.36 (0.04–0.68), p = 0.027	0.24 (0.07–0.41), p = 0.006	0.05 (-0.03–0.14), p = 0.209	0.07 (-0.19–0.34), p = 0.591
Female provider	-0.29 (-0.98–0.40), p = 0.408	0.16 (-0.20–0.53), p = 0.376	-0.03 (-0.22–0.15), p = 0.708	-0.45 (-1.02–0.12), p = 0.122 = 1
Bonus (vs fixed salary)	2.09 (1.34–2.84), p < 0.001	0.78 (0.39–1.17), p < 0.001	0.35 (0.16–0.55), p < 0.001	0.96 (0.35–1.57), p = 0.002
% of 3 most qualified clinicians who are doctors	4.31 (2.63–5.99), p < 0.001	3.90 (3.03–4.77) p < 0.001	-0.02 (-0.47–0.42), p = 0.917	0.25 (-1.12–1.62), p = 0.4716
Base model + provider characteristics + facility characteristics				
IRT effort	0.30 (-0.01–0.62), p = 0.057	0.15 (-0.10–0.32), p = 0.066	0.05 (-0.04–0.14), p = 0.267	0.11 (-0.15–0.38), p = 0.403
Female provider	-0.07 (-0.74–0.60) p = 0.844	0.25 (-0.10–0.60), p = 0.162	-0.03 (-0.22–0.15), p = 0.717	-0.34 (-0.90–0.23), p = 0.244
Bonus (vs fixed salary)	1.50 (0.74–2.26), p < 0.001	0.45 (0.06–0.84), p = 0.024	0.32 (0.11–0.52), p = 0.003	0.71 (0.07–1.34), p = 0.029
% of 3 most qualified clinicians who are doctors	3.18 (1.47–4.90), < 0.001	3.28 (2.39–4.16), p < 0.001	-0.13 (-0.60–0.34), p = 0.659	-0.20 (-1.63–1.23), p = 0.782
Hospital (vs dispensary)	1.29 (0.34–2.24), 0.008	1.14 (0.64–01.64), p < 0.001	0.09 (-0.18–0.35), p = 0.517	-0.09 (-0.72–0.89), p = 0.834
Health centre (vs dispensary)	0.58 (-0.16–1.31), 0.122	0.53 (0.14–0.92), p = 0.008	0.22 (0.01–0.42), p = 0.035	0.01 (-0.62–0.63), p = 0.834
For-profit (vs not-for-profit)	1.68 (0.90–2.45), p < 0.001	0.74 (0.33–1.15), p < 0.001	0.16 (-0.05–0.37), p = 0.136	0.95 (0.29–1.61), p = 0.005
Peri-urban (vs rural)	0.25 (-0.53–1.03), p = 0.529	-0.10 (-0.51–0.31), p = 0.626	0.01 (-0.20–0.23), p = 0.894	0.42 (-0.23–1.08), p = 0.206
Urban (vs rural)	1.05 (0.26–1.84), p = 0.010	0.54 (0.13–0.95), p = 0.010	0.03 (-0.18–0.25), p = 0.771	0.47 (-0.20–1.13), p = 0.167
Any insurance revenue	0.29 (-0.42–1.00), p = 0.420	0.42 (0.04–0.79), p = 0.029	-0.01 (-0.21–0.18), p = 0.902	-0.10 (-0.70–0.50), p = 0.750

Coefficients are from linear regression models. Base model includes adjustment for SP fixed effects, SP case and SafeCare intervention arm

Provider effort was associated with higher total fees, with an increase of USD 0.51 in fees paid per one standard deviation increase in effort IRT score (p = 0.003, Table 7). Most of this increase was explained by higher consultation fees, which had an increase of USD 0.37 for each standard deviation increase in effort IRT score (p < 0.001). When adjusting for provider and facility characteristics, the effect of effort on fees was somewhat attenuated, with a one standard deviation increase in effort associated with a USD 0.30 increase in the overall fee (p = 0.057), and a USD 0.15 increase in the consultation fee (p = 0.016).

Bonus or revenue-based payments for outpatient clinicians increased mean fees by USD 1.50 (p < 0.001), and this acted jointly through increases in the consultation fee (USD 0.45, p = 0.024), lab fee (USD 0.32, p = 0.003) and drug fee (USD 0.71, p = 0.029). Mean fees were USD 1.68 higher in for-profit than not-for-profit facilities (p < 0.001), and this acted through both higher consultation fees (USD 0.74, p< 0.001) and drug fees (USD 0.95, p = 0.005).

Fees at facilities with at least three doctors on the outpatient staff were USD 3.18 higher than those without any doctors (p < 0.001), and this acted solely through the consultation fee, which was USD 3.28 higher (p < 0.001). Hospitals charged an average of USD 1.29 more in fees than dispensaries (p = 0.008), again driven by the consultation fee, which was USD 1.14 higher (p < 0.001). Fees were higher in urban than rural facilities (USD 1.05, p = 0.010) and this acted though the consultation fee (USD 0.54, p = 0.010).

## Discussion

Only 1 in 18 asthma SPs received the correct management (prescription of inhaler) and only 1 in 4 TB SPs were correctly referred for testing. Unnecessary care was widespread: three-quarters of all SPs received at least one unnecessary drug or test. In general, provider effort was low, with clinicians carrying out around one third of recommended checklist items. Increased effort in the consultation was strongly associated with an increased likelihood of correct care and a decrease in unnecessary care. This suggests that providers who exert more effort are not simply providing ‘more of everything’ but that perhaps they are being more precise in their diagnosis, with the increased history taking and physical exams enabling them to avoid providing unnecessary care. It is worth noting that given the average correct management rate was only 15%, the 81% increase in the chance of correct management associated with a one standard deviation increase in effort is a small absolute effect, and the chance of receiving correct care remained low.

The qualification level of outpatient staff was not an independent predictor of either quality of care outcome in the multivariate model, when controlling for provider effort, but it was strongly correlated with provider effort itself. It may be through exerting greater effort that higher qualified providers are able to deliver better quality of care. Provider payment mechanism was also important: those working at facilities where they were paid a bonus or share of revenue were more likely to provide unnecessary care than at facilities which paid a fixed salary. This suggests that such financial incentives may in fact be detrimental to patient care, increasing the likelihood of unnecessary care without increasing the likelihood of correct care.

For-profit facilities were much less likely to provider correct care, though no more likely to provide unnecessary care, than not-for-profit facilities. At first glance this runs contrary to assumptions about profit-making facilities: there is no clear reason for profit to incentivise poor care, unless it is through providing more unnecessary care. However, it is worth noting that correct management of TB was ordering a sputum test, or referring to a facility which could do a sputum test. Many small facilities do not have the capacity to do the test, so for for-profit facilities a referral would mean losing the income generated from treating the patient otherwise.

Factors associated with an increased consultation fee (without any increase in fees for tests or drugs) seemed to be related to skill and level: more effort, more outpatient clinicians being fully qualified doctors, hospitals and health centres (vs dispensaries) and urban facilities compared to rural ones. This may be a case of more skilful providers signalling their higher quality of care through the fee for the initial consultation, or that those which charge a substantial consultation fee feel the need to justify it through exerting more effort, or are incentivised to do so. The characteristics associated with higher consultation, lab and drugs fees were profit status and provider payment mechanism. This may be the result of both higher fixed prices and the incentive to sell additional unnecessary tests and drugs in for-profit facilities, and those which pay clinicians a share of revenue or bonus, rather than a fixed salary. It is notable that not-for-profit facilities are more likely to provide correct care while also delivering it at a lower cost, though without additional effort. This may be because not-for-profit facilities in this sample are more likely to be higher-level hospitals and health centres, with better qualified staff [7].

The use of SPs has a number of strengths. Unlike record extraction, we know the correct diagnosis and treatment for every SP, so correct and unnecessary care can be measured precisely and directly. We can measure effort through recording whether history questions were asked, whereas a medical record may only contain a brief summary of the information gathered, not the full list of questions asked. Using standardised patients removes the risk of case-mix and patient-mix bias, as all providers deal with the same comparable condition. Compared to direct observation, it removes the Hawthorne effect, whereby providers alter their behaviour because they know that they are being observed, and compared to patient exit interview it removes recall bias.

The study also has limitations. For safety reasons, our SPs did not do all recommended tests or buy certain types of drugs (such as injections) which may both have reduced the overall fees payable as well as affected the provider’s ability to make a diagnosis (though cases were designed such that tests were not required to make the correct diagnosis and provide correct management). Effort is operationalised as a function of questions and physical exams in the consultation, but this is an imperfect and indirect proxy measure, as the measure will also be a function of the provider’s skill and knowledge. The effort measure also cannot take account of how well actions are carried out: a provider who takes several minutes to listen carefully to breathing on the front and back of the chest is rated the same as one who listens only briefly without paying much attention.

Our findings are in line with other SP studies in India (rural Madhya Pradesh [17] and West Bengal [16], and urban Mumbai, Patna & Delhi [28]), China [28] and Senegal [27], which have shown that when providers make more effort, they are more likely to provide correct care. Most of those studies were among private providers only, except the one set in China which included only public providers and in Madhya Pradesh which included both. The only study we have identified where effort did not predict correct management was in Kenya [34], where the result was driven by providers correctly referring TB SPs for testing despite asking very few questions [28]. The authors of that study suggest that effort does not improve management in that setting because of clear protocols to refer patients with persistent cough for TB testing, in contrast to our findings that effort in the consultation was important for correct management. In the Kenyan study, 50% of TB SPs were correctly referred, and were asked an average of 42% of the recommended nine history questions (a mean of 3.8 questions) [34], whereas we observed correct referral of only 25%, but a mean of 10.1 checklist items completed. This suggests that the role of effort may be less important than training, messaging and protocols for providers. The difference may also be explained by sector: the Kenyan study included both public and private providers.

There is mixed evidence on whether provider effort is protective against unnecessary care. Two SPs studies in rural India found no association between effort and unnecessary treatment, despite effort predicting correct management [16, 17]. Another study among public and private doctors in Delhi found that providers who made more effort prescribed more drugs, though no attempt was made to classify them as unnecessary [23]. However, an SP study in China found that increased effort was associated with reduced use of unnecessary antibiotics [18], more in line with our own findings, with the authors suggesting diagnostic uncertainty as a key driver of inappropriate antibiotic use. The variation in results across settings suggests the reasons behind the provision of unnecessary care are context-specific, and may not be able to be tackled with the same tools in different places.

Interventions to encourage providers to exert more effort may both increase correct care and reduce unnecessary care, allowing the health system to operate more efficiently. One way to do this is through training: a randomized controlled trial of a training programme for informal providers in India found a positive effect on effort after nine months (though in this study, increased effort did not decrease unnecessary care) [16]. However, this kind of training may need to be carefully targeted at individual providers; a randomized controlled trial of a broader facility level quality improvement programme in Tanzania did not increase provider effort, or improve correct management [29].

To reduce unnecessary care and inflated fees, addressing payment structures and provider incentives may be more important than training. Facilities could be mandated to pay providers only using a fixed salary, with bonuses based on facility profits or volume of patients outlawed by regulatory mechanisms. However, this would not address incentives where the provider is also the owner of the business. Further steps could include a requirement that all prescribed medicines are dispensed by an independent pharmacy, or diagnostic tests carried out by independent labs, though more intensive regulatory intervention would be required to ensure compliance. Given the expansion of social health insurance programmes, strategic purchasing arrangements by private or public insurers, such as capitation or reimbursement based on diagnostic related groups, may play an important role in preventing unnecessary care in the future.

## Conclusion

In this standardised patient study in Tanzanian private health facilities, we have added to evidence that a provider exerting greater effort increases the likelihood of giving the correct management for a patient’s condition, and made the novel finding that it reduces unnecessary care. This points towards potential interventions to tackle overprovision. Another novel finding is that providers who make more effort charge higher fees, which may be a way to signal their quality of care.

## Electronic supplementary material

Below is the link to the electronic supplementary material.

Supplementary Material 1

## Data Availability

The datasets used in this study are available from the corresponding author on reasonable request.

## List of Abbreviations

IRT: Item response theory
LMICs: Low- and middle-income countries
SP: Standardised patient
