# Supplementary material for: Does increased provider effort improve quality of care? Evidence from a standardised patient study on correct and unnecessary treatment
Source: BMC Health Serv Res. 2023 Feb 23;23:190. doi: 10.1186/s12913-023-09149-5 (PMC9948477; doi:10.1186/s12913-023-09149-5)
Supplement: Supplementary file 1 — Supplementary Material 1 [file 12913_2023_9149_MOESM1_ESM.docx]

## Item response theory effort measure construction

Item response theory (IRT) assumes the existence of a latent variable,$\theta$, in this case provider effort. Whether or not the provider carries out each of the items on the checklist of history taking and physical exams is assumed to be a function of this latent variable. IRT relies on four key assumptions:

1. Monotonicity: that as the latent trait score (provider effort) increases, the probability of carrying out each checklist action also increases
2. Unidimensionality: that checklist actions measure just one latent trait
3. Independence: that the probability of carrying out one checklist action does not depend on whether or not another action in the list was carried out
4. Invariance: the probability of carrying out a given checklist action is the same for different providers who have equal effort scores

The latent variable is modelled through an item characteristic curve (ICC) for the probability of completing each checklist item as a function of the latent variable: the ICC for item$i$ can be thought of as $P_{i}(\theta)$. The ICC is modelled using a two-parameter logistic model, where the binary outcome is whether or not the item was completed, as a function of $\theta$, and two parameters which can vary by item, $a_{i}$ and $b_{i}$:

$$P_{i}\left( \theta\right)=\frac{1}{1+e^{-a_{i}(\theta-b_{i})}}$$

Parameter $a_{i}$ is the discrimination parameter, which is proportional to the maximum slope of the ICC, and can be thought of as measuring the ability of item $i$ to distinguish between values of $\theta$. Parameter $b_{i}$ is the difficulty parameter, and is equal to the value of $\theta$ where the probability of carrying out item $i$ is 0.5.

The discrimination and difficulty parameters are estimated by multiplying the ICCs for every item $i$ together to produce a likelihood function, then fitting the model using maximum likelihood estimation within Stata. The distribution of $\theta$was estimated separately for TB and asthma cases, based on the separate checklists, then standardised to produce effort scores with mean 0 and standard deviation 1. The frequency, discrimination and difficulty coefficients for each item are given below.

The frequency of each item, and its discrimination and difficulty coefficients (and p-values for coefficients being non-zero) are given in Table A1. For asthma, the actions completed most frequently were asking age (86%), about the nature or type of breathing difficulty (79%) and whether the SP had chest pain (72%). All other actions were completed in less than 70% of consultations. The least frequently completed actions, carried out in less than 1% of consultations, were asking about recent weight loss, if the SP was breathless at rest during attacks, and what distance they could walk during attacks. In the IRT analysis, items with high discrimination coefficients which were significant at the 5% level included asking if the SP had eaten any new food, about the circumstances of their attack and the time of day of their symptoms. Items with high difficulty coefficients which were significant at the 5% level included examining the throat, and asking about recent weight loss or if the SP was breathless at rest during attacks.

For TB, the actions completed most frequently were asking age (97%), about the duration or date of onset of coughing (90%) and whether cough produced mucus or sputum (81%). All other actions were completed in less than 81% of consultations. The least frequently completed actions, carried out in less than 5% of consultations, were asking about whether the SP was a smoker, drank alcohol or had diabetes. In the IRT analysis, items with the highest discrimination coefficients included asking if the SP had anyone in their family with TB or a persistent cough, or contact with anyone else with TB. Items with high difficulty coefficients which were significant at the 5% level included asking about loss of appetite, wheezing, or if anyone in the family had a persistent cough. For both asthma and TB, graphs showing the item characteristic curves for the items with the lowest, median and highest difficulty and discrimination are given in the appendix, along with the test characteristic curves showing the relationship between IRT score and number of items completed.

| Table A1: components of effort score | | | | | | | | | | |
| --- | --- | --- | --- | --- | --- | --- | --- | --- | --- | --- |
|  | Asthma | | | | | TB | | | | |
| Item | Frequency (%) | Discrimination | | Difficulty | | Frequency (%) | Discrimination | | Difficulty | |
|  |  | Coefficient | p | Coefficient | p |  | Coefficient | p | Coefficient | p |
| Physical exams (both cases) |  |  |  |  |  |  |  |  |  |  |
| Throat examined | 4.4 | 0.80 | 0.056 | 4.21 | 0.027 | 8.0 | 0.31 | 0.268 | 7.91 | 0.256 |
| Pulse taken | 33.9 | 0.49 | 0.010 | 1.43 | 0.014 | 25.7 | 0.28 | 0.118 | 3.86 | 0.115 |
| Blood pressure taken | 55.5 | 0.60 | 0.002 | -0.39 | 0.134 | 37.2 | 0.39 | 0.02 | 1.39 | 0.037 |
| Temperature taken with thermometer | 19.4 | 0.31 | 0.138 | 4.69 | 0.129 | 29.2 | 0.24 | 0.155 | 3.71 | 0.156 |
| Listened to chest with stethoscope | 51.1 | 0.05 | 0.760 | -0.92 | 0.822 | 43.8 | 0.10 | 0.512 | 2.50 | 0.534 |
| Symptoms (both cases) |  |  |  |  |  |  |  |  |  |  |
| Time of day of symptoms | 39.2 | 1.33 | <0.001 | 0.45 | 0.002 | 30.5 | 0.82 | <0.001 | 1.14 | <0.001 |
| Any wheezing | 31.3 | 0.56 | 0.004 | 1.51 | 0.005 | 5.3 | 1.88 | <0.001 | 2.22 | <0.001 |
| Recent weight loss | 0.9 | 2.06 | 0.106 | 3.18 | 0.003 | 22.6 | 1.37 | <0.001 | 1.20 | <0.001 |
| Night sweats | 7.0 | 1.06 | 0.005 | 2.84 | <0.001 | 46.5 | 2.09 | <0.001 | 0.13 | 0.244 |
| Coughing up mucus/sputum | 32.2 | 0.76 | <0.001 | 1.11 | 0.001 | 80.5 | 1.96 | <0.001 | -1.15 | <0.001 |
| Chest pain | 71.8 | 1.08 | <0.001 | -1.06 | <0.001 | 53.5 | 0.76 | <0.001 | -0.21 | 0.305 |
| Fevers | 46.3 | 0.18 | 0.250 | 0.83 | 0.417 | 73.5 | 1.49 | <0.001 | -0.95 | <0.001 |
| Other history (both cases) |  |  |  |  |  |  |  |  |  |  |
| Previous careseeking /medication | 52.0 | 0.55 | 0.002 | -0.15 | 0.562 | 77.4 | 0.70 | 0.002 | -1.95 | 0.001 |
| Smoker | 8.8 | 1.08 | 0.003 | 2.57 | <0.001 | 4.9 | 0.50 | 0.166 | 6.21 | 0.141 |
| Age | 86.3 | 0.16 | 0.471 | -11.37 | 0.468 | 97.3 | 0.45 | 0.345 | -8.30 | 0.323 |
| Occupation | 37.0 | 0.29 | 0.078 | 1.85 | 0.097 | 28.3 | 0.31 | 0.072 | 3.07 | 0.072 |
| Symptoms (asthma) |  |  |  |  |  |  |  |  |  |  |
| Breathless at rest during attack | 0.9 | 2.03 | 0.083 | 3.21 | 0.002 |  |  |  |  |  |
| What distance can you walk during an attack | 0.4 | 0.27 | 0.817 | 20.07 | 0.815 |  |  |  |  |  |
| Keeps awake at night | 2.2 | 0.85 | 0.137 | 4.86 | 0.084 |  |  |  |  |  |
| Circumstances of recent attack/ what were you doing | 48.5 | 1.41 | <0.001 | 0.07 | 0.602 |  |  |  |  |  |
| Length of attack | 30.4 | 1.09 | <0.001 | 0.94 | <0.001 |  |  |  |  |  |
| Frequency of attacks | 48.5 | 0.53 | 0.003 | 0.13 | 0.640 |  |  |  |  |  |
| Shortness of breath constant or episodic | 35.2 | 0.71 | <0.001 | 0.95 | 0.002 |  |  |  |  |  |
| Any triggers for attacks | 34.4 | 0.79 | <0.001 | 0.93 | 0.001 |  |  |  |  |  |
| Any previous attacks | 31.3 | 0.89 | <0.001 | 1.03 | <0.001 |  |  |  |  |  |
| Type of breathing difficulty | 79.3 | 0.66 | 0.003 | -2.25 | 0.001 |  |  |  |  |  |
| Date of onset of attacks | 33.0 | 0.60 | 0.002 | 1.27 | 0.003 |  |  |  |  |  |
| Does anything improve attack/how to cope | 26.4 | 0.90 | <0.001 | 1.33 | <0.001 |  |  |  |  |  |
| Other history (asthma) |  |  |  |  |  |  |  |  |  |  |
| Allergies | 16.7 | 1.06 | <0.001 | 1.82 | <0.001 |  |  |  |  |  |
| Childhood asthma or similar attacks | 8.8 | 0.92 | 0.005 | 2.90 | 0.001 |  |  |  |  |  |
| Family history of asthma | 40.5 | 0.99 | <0.001 | 0.47 | 0.009 |  |  |  |  |  |
| Asthmatic/previous diagnosis of asthma | 67.8 | 0.62 | 0.001 | -1.30 | 0.002 |  |  |  |  |  |
| Any new/unusual foods | 8.8 | 2.16 | <0.001 | 1.74 | <0.001 |  |  |  |  |  |
| Symptoms (TB) |  |  |  |  |  |  |  |  |  |  |
| Duration/onset of coughing |  |  |  |  |  | 89.8 | 1.31 | <0.001 | -2.13 | <0.001 |
| Blood in mucus/sputum |  |  |  |  |  | 26.1 | 1.05 | <0.001 | 1.21 | <0.001 |
| Loss of appetite |  |  |  |  |  | 14.1 | 0.80 | 0.001 | 2.51 | <0.001 |
| Breathing difficulty /shortness of breath |  |  |  |  |  | 23.9 | 0.92 | <0.001 | 1.47 | <0.001 |
| Other history (TB) |  |  |  |  |  |  |  |  |  |  |
| Contact with anyone with TB |  |  |  |  |  | 15.9 | 3.28 | <0.001 | 1.13 | <0.001 |
| Previous TB |  |  |  |  |  | 11.9 | 2.31 | <0.001 | 1.47 | <0.001 |
| Anyone in family with TB |  |  |  |  |  | 13.3 | 2.63 | <0.001 | 1.34 | <0.001 |
| Anyone in family with persistent cough |  |  |  |  |  | 6.6 | 2.68 | <0.001 | 1.80 | <0.001 |
| Drinker |  |  |  |  |  | 3.5 | -0.02 | 0.955 | -144.78 | 0.955 |
| Diabetic |  |  |  |  |  | 1.3 | 0.63 | 0.342 | 7.15 | 0.304 |
| HIV status |  |  |  |  |  | 21.2 | 1.03 | <0.001 | 1.53 | <0.001 |
| Type/name of medication taken |  |  |  |  |  | 72.6 | 0.67 | 0.002 | -1.60 | 0.001 |
| Course length /duration of taking medication |  |  |  |  |  | 48.2 | 0.62 | 0.001 | 0.13 | 0.592 |

## Referral sensitivity analysis

Table A2: Comparing referral and non-referral in correctly managed TB SPs

|  | n | % received unnecessary care (95% CI) | Test for equality of proportions |
| --- | --- | --- | --- |
| Referred | 17 | 52.9 (29.2-76.7) | p=0.33 |
| Not referred | 39 | 66.7 (51.9- 81.5) |  |

Table A3: Result on unnecessary care excluding referrals

|  | Any unnecessary care |
| --- | --- |
|  | Relative risk |
| Base model |  |
| IRT effort | 0.92 (0.87– 0.97), p=0.003 |
| Base model + provider characteristics |  |
| IRT effort | 0.93 (0.88 – 0.99), p =0.007 |
| HCW female | 0.89 (0.78 – 1.02), p=0.092 |
| Bonus (vs fixed salary) | 1.25 (1.12 – 1.39), p<0.001 |
| % of clinicians doctors | 0.80 (0.58 – 1.10) p=0.165 |
| Base model + provider characteristics + facility characteristics |  |
| IRT effort | 0.94 (0.89 – 0.99),p =0.022 |
| HCW female | 0.88 (0.77 – 1.01), p=0.066 |
| Bonus (vs fixed salary) | 1.25 (1.10 – 1.40), p<0.001 |
| % of clinicians doctors | 0.77 (0.58– 1.07) p=0.116 |
| Hospital (vs dispensary) | 0.89 (0.74 – 1.08), p=0.227 |
| Health centre (vs dispensary) | 1.02 (0.90 – 1.16), p=0.772 |
| For profit (vs not for profit) | 1.06 (0.92 – 1.22), p=0.457 |
| Peri-urban (vs rural) | 1.07 0.92 – 1.24), p=0.363 |
| Urban (vs rural) | 0.95 (0.81 – 1.12), p=0.548 |
| Any insurance revenue | 1.03 (0.90 – 1.17), p=0.686 |

## Follow-up sensitivity analysis

Table A4: Comparing outcomes by follow up

|  | n | % received correct management (95% CI) | Test for equality of proportions | % received unnecessary care (95% CI) | Test for equality of proportions |
| --- | --- | --- | --- | --- | --- |
| Asked to return | 135 | 13.3 (7.6-19.1) | p=0.4715 | 62.2 (54.0 – 70.4) | P=0.001 |
| Not asked to return | 319 | 16.0 (12.0- 20.0) |  | 79.3 (74.9-83.8) |  |

## Fees modelling with outcomes

| Table A5: Linear regression models for fees with quality of care outcomes as independent variables | | | | |
| --- | --- | --- | --- | --- |
| Base model + provider characteristics + facility characteristics + quality of care outcomes | Total fee | Consultation fee | Lab fee | Medicines fee |
| IRT effort | 0.55 (0.25– 0.85), p<0.001 | 0.19 (0.02 – 0.36), p=0.025 | 0.65 (-0.03 – 0.15), p=0.284 | 0.31 (0.06 – 0.59), p=0.015 |
| HCW female | 0.19 (-0.43 – 0.82) p=0.543 | 0.28 (-0.07 – 0.63), p=0.117 | -0.01 (-0.20 – 0.17), p=0.873 | -0.10 (-0.61 – 0.42), p=0.717 |
| Bonus (vs fixed salary) | 1.36 (0.65 – 2.07), p<0.001 | 0.51 (0.12 – 0.91), p=0.010 | 0.27 (0.06 – 0.548), p=0.01 | 0.54 (-0.05 – 1.12), p=0.071 |
| % of clinicians doctors | 3.55 (1.95 – 5.15), p<0.001 | 3.25 (2.37 – 4.13), p<0.001 | -0.13 (-0.60 – 0.34), p=0.762 | -0.11 (-1.20 – 1.41), p=0.872 |
| Hospital (vs dispensary) | 1.55 (0.66 – 2.44), p=0.001 | 1.17 (0.68 – 1.67), p<0.001 | 0.10 (-0.16 – 0.36), p=0.444 | 0.29 (-0.45 – 1.03), p=0.444 |
| Health centre (vs dispensary) | 0.60 (-0.08 – 1.28), 0.086 | 0.56 (0.17 – 0.95), p=0.005 | 0.20 (0.01 – 0.40), p=0.044 | -0.02 (-0.59 – 0.55), p=0.950 |
| For profit | 1.53 (0.81 – 2.26), p<0.001 | 0.70 (0.29 – 1.11), p=0.001 | 0.16 (-0.05 – 0.37), p=0.141 | 0.82 (0.22 – 1.43), p=0.008 |
| peri-urban (vs rural) | 0.20 (-0.53 – 0.92), p=0.595 | -0.04 (-0.44 – 0.37), p=0.851 | -0.02 (-0.23 – 0.19), p=0.844 | 0.32 (-0.29 – 0.92), p=0.303 |
| urban (vs rural) | 1.04 (0.30 – 1.78), p=0.006 | 0.53 (0.13 – 0.94), p=0.010 | 0.03 (-0.18 – 0.25), p=0.764 | 0.44 (-0.17 – 1.04), p=0.157 |
| Any insurance revenue | 0.34 (-0.32 – 1.00), p=0.308 | 0.45 (0.08 – 0.82), p=0.017 | -0.02 (-0.21 – 0.17), p=0.830 | -0.05 (-0.60 – 0.50), p=0.862 |
| Correct management | -1.39 (-2.21 – 2.89), p=0.001 | -0.65 (-1.10 - -0.21), p=0.004 | 0.13 (-0.11 – 0.38), p=0.284 | -0.92 (-1.59 - -0.25), p=0.007 |
| Any unnecessary care | 2.24 (1.59 -2.89), p<0.001 | -0.27 (-0.63 – 0.09), p=0.137 | 0.40 (0.21 – 0.59), p<0.001 | 2.14 (1.61 – 2.67), p<0.001 |

## Outcomes dichotomised by consultation fee

| Table A6: Consultation outcomes, effort, and fees paid | | | |
| --- | --- | --- | --- |
|  | High (>1USD) consultation fee (n=186) | Low (<1USD) consultation fee (n=241) | |
| Outcome of consultation |  |  | |
| Correct management | 0.19 (0.39) | 0.13 (0.34) | |
| Unnecessary care | 0.72 (0.45) | 0.74 (0.44) | |
|  |  |  | |
| Provider effort |  |  | |
| Number of checklist items carried out¹ | 11.74 (4.12) | 9.73 (4.01) | |
| Proportion of checklist terms carried out | 0.38 (0.13) | 0.31 (0.13) | |
|  |  |  | |
| Fees paid |  |  | |
| Total fee USD | 5.69 (3.93) | 3.34 (2.65) | |
| Consultation fee | 2.49 (2.23) | 0.46 (0.38) | |
| Diagnostic tests fees | 0.41 (1.08) | 0.25 (0.61) | |
| Medicines fees | 2.86 (2.88) | 2.61 (2.42) | |
| ¹Target number of checklist items was 33 for asthma and 29 for TB | | |  |

## Unnecessary drugs and tests

| Table A7: Unnecessary drugs by ATC classification | | |  |
| --- | --- | --- | --- |
| ATC code | ATC level 2 | ATC level 3 (antibiotics only) | n |
| A02BC05 | DRUGS FOR ACID RELATED DISORDERS | 1 | |
| A03BA03 | DRUGS FOR FUNCTIONAL GASTROINTESTINAL DISORDERS | 1 | |
| A07AA02 | ANTIDIARRHEALS, INTESTINAL ANTIINFLAMMATORY/ANTIINFECTIVE AGENTS | 1 | |
| C03CA01 | DIURETICS |  | 4 |
| C07AB03 | BETA BLOCKING AGENTS |  | 1 |
| C08CA01 | CALCIUM CHANNEL BLOCKERS | 1 | |
| C08CA05 | CALCIUM CHANNEL BLOCKERS | 1 | |
| C09AA01 | AGENTS ACTING ON THE RENIN-ANGIOTENSIN SYSTEM | 2 | |
| C09CA01 | AGENTS ACTING ON THE RENIN-ANGIOTENSIN SYSTEM | 1 | |
| C10AA05 | LIPID MODIFYING AGENTS |  | 1 |
| H02AB02 | CORTICOSTEROIDS FOR SYSTEMIC USE | 1 | |
| H02AB06 | CORTICOSTEROIDS FOR SYSTEMIC USE | 26 | |
| H02AB09 | CORTICOSTEROIDS FOR SYSTEMIC USE | 1 | |
| J01AA02 | ANTIBACTERIALS FOR SYSTEMIC USE | TETRACYCLINES | 6 |
| J01BA01 | ANTIBACTERIALS FOR SYSTEMIC USE | AMPHENICOLS | 4 |
| J01CA01 | ANTIBACTERIALS FOR SYSTEMIC USE | BETA-LACTAM ANTIBACTERIALS, PENICILLINS | 2 |
| J01CA04 | ANTIBACTERIALS FOR SYSTEMIC USE | BETA-LACTAM ANTIBACTERIALS, PENICILLINS | 48 |
| J01CA51 | ANTIBACTERIALS FOR SYSTEMIC USE | BETA-LACTAM ANTIBACTERIALS, PENICILLINS | 103 |
| J01CE01 | ANTIBACTERIALS FOR SYSTEMIC USE | BETA-LACTAM ANTIBACTERIALS, PENICILLINS | 13 |
| J01CE02 | ANTIBACTERIALS FOR SYSTEMIC USE | BETA-LACTAM ANTIBACTERIALS, PENICILLINS | 5 |
| J01CR02 | ANTIBACTERIALS FOR SYSTEMIC USE | BETA-LACTAM ANTIBACTERIALS, PENICILLINS | 12 |
| J01CR50 | ANTIBACTERIALS FOR SYSTEMIC USE | BETA-LACTAM ANTIBACTERIALS, PENICILLINS | 8 |
| J01DB01 | ANTIBACTERIALS FOR SYSTEMIC USE | OTHER BETA-LACTAM ANTIBACTERIALS | 16 |
| J01EE01 | ANTIBACTERIALS FOR SYSTEMIC USE | SULFONAMIDES AND TRIMETHOPRIM | 21 |
| J01FA01 | ANTIBACTERIALS FOR SYSTEMIC USE | MACROLIDES, LINCOSAMIDES AND STREPTOGRAMINS | 22 |
| J01FA09 | ANTIBACTERIALS FOR SYSTEMIC USE | MACROLIDES, LINCOSAMIDES AND STREPTOGRAMINS | 1 |
| J01FA10 | ANTIBACTERIALS FOR SYSTEMIC USE | MACROLIDES, LINCOSAMIDES AND STREPTOGRAMINS | 19 |
| J01GB03 | ANTIBACTERIALS FOR SYSTEMIC USE | AMINOGLYCOSIDE ANTIBACTERIALS | 2 |
| J01MA02 | ANTIBACTERIALS FOR SYSTEMIC USE | QUINOLONE ANTIBACTERIALS | 5 |
| J01MA06 | ANTIBACTERIALS FOR SYSTEMIC USE | QUINOLONE ANTIBACTERIALS | 1 |
| J01MA12 | ANTIBACTERIALS FOR SYSTEMIC USE | QUINOLONE ANTIBACTERIALS | 2 |
| J01RA13 | ANTIBACTERIALS FOR SYSTEMIC USE | COMBINATIONS OF ANTIBACTERIALS | 2 |
| J01XD01 | ANTIBACTERIALS FOR SYSTEMIC USE | OTHER ANTIBACTERIALS | 6 |
| J01XD02 | ANTIBACTERIALS FOR SYSTEMIC USE | OTHER ANTIBACTERIALS | 2 |
| J01XE01 | ANTIBACTERIALS FOR SYSTEMIC USE | OTHER ANTIBACTERIALS | 1 |
| M01AB05 | ANTIINFLAMMATORY AND ANTIRHEUMATIC PRODUCTS | 2 | |
| M01AB16 | ANTIINFLAMMATORY AND ANTIRHEUMATIC PRODUCTS | 1 | |
| M01AB55 | ANTIINFLAMMATORY AND ANTIRHEUMATIC PRODUCTS | 1 | |
| M01AC01 | ANTIINFLAMMATORY AND ANTIRHEUMATIC PRODUCTS | 3 | |
| M01AC06 | ANTIINFLAMMATORY AND ANTIRHEUMATIC PRODUCTS | 1 | |
| M01AE01 | ANTIINFLAMMATORY AND ANTIRHEUMATIC PRODUCTS | 3 | |
| N02AX02 | ANALGESICS |  | 2 |
| N02BA51 | ANALGESICS |  | 1 |
| N02BE01 | ANALGESICS |  | 39 |
| N05BA01 | PSYCHOLEPTICS |  | 4 |
| P01BD51 | ANTIPROTOZOALS |  | 2 |
| P01BF01 | ANTIPROTOZOALS |  | 5 |
| P01BF07 | ANTIPROTOZOALS |  | 1 |
| P02CA01 | ANTHELMINTICS |  | 4 |
| P02CA03 | ANTHELMINTICS |  | 18 |
| P02CE01 | ANTHELMINTICS |  | 1 |
| R03AC02 | DRUGS FOR OBSTRUCTIVE AIRWAY DISEASES | 7 | |
| R03DA05 | DRUGS FOR OBSTRUCTIVE AIRWAY DISEASES | 2 | |
| R06AB02 | ANTIHISTAMINES FOR SYSTEMIC USE | 18 | |
| R06AB52 | ANTIHISTAMINES FOR SYSTEMIC USE | 1 | |
| R06AD02 | ANTIHISTAMINES FOR SYSTEMIC USE | 1 | |
| R06AE07 | ANTIHISTAMINES FOR SYSTEMIC USE | 19 | |
| R06AX02 | ANTIHISTAMINES FOR SYSTEMIC USE | 1 | |
| R06AX13 | ANTIHISTAMINES FOR SYSTEMIC USE | 2 | |
| R06AX17 | ANTIHISTAMINES FOR SYSTEMIC USE | 1 | |
| R06AX26 | ANTIHISTAMINES FOR SYSTEMIC USE | 1 | |
| UNSPECIFIED |  |  | 30 |
| UNSPECIFIED COUGH SYRUP | |  | 11 |
| UNSPECIFIED NASAL SPRAY | |  | 1 |
| UNSPECIFIED ORS | |  | 1 |
| UNSPECIFIED VITAMINS | |  | 7 |

| Table A8: Unnecessary drugs grouped by ATC Level 2 (3 for antibiotics) |  |
| --- | --- |
| Drug group | n |
| BETA-LACTAM ANTIBACTERIALS, PENICILLINS | 191 |
| ANTIHISTAMINES FOR SYSTEMIC USE | 44 |
| MACROLIDES, LINCOSAMIDES AND STREPTOGRAMINS | 42 |
| ANALGESICS | 42 |
| UNSPECIFIED | 30 |
| CORTICOSTEROIDS FOR SYSTEMIC USE | 28 |
| ANTHELMINTICS | 23 |
| SULFONAMIDES AND TRIMETHOPRIM | 21 |
| OTHER BETA-LACTAM ANTIBACTERIALS | 16 |
| ANTIINFLAMMATORY AND ANTIRHEUMATIC PRODUCTS | 11 |
| UNSPECIFIED COUGH SYRUP | 11 |
| OTHER ANTIBACTERIALS | 9 |
| DRUGS FOR OBSTRUCTIVE AIRWAY DISEASES | 9 |
| QUINOLONE ANTIBACTERIALS | 8 |
| ANTIPROTOZOALS | 8 |
| UNSPECIFIED VITAMINS | 7 |
| TETRACYCLINES | 6 |
| DIURETICS | 4 |
| AMPHENICOLS | 4 |
| PSYCHOLEPTICS | 4 |
| AGENTS ACTING ON THE RENIN-ANGIOTENSIN SYSTEM | 3 |
| CALCIUM CHANNEL BLOCKERS | 2 |
| AMINOGLYCOSIDE ANTIBACTERIALS | 2 |
| COMBINATIONS OF ANTIBACTERIALS | 2 |
| UNSPECIFIED NASAL SPRAY | 1 |
| DRUGS FOR ACID RELATED DISORDERS | 1 |
| DRUGS FOR FUNCTIONAL GASTROINTESTINAL DISORDERS | 1 |
| ANTIDIARRHEALS, INTESTINAL ANTIINFLAMMATORY/ANTIINFECTIVE AGENTS | 1 |
| BETA BLOCKING AGENTS | 1 |
| LIPID MODIFYING AGENTS | 1 |
| UNSPECIFIED ORS | 1 |

| Table A9: Unnecessary tests ordered | |
| --- | --- |
| Test type | n |
| Urinalysis | 33 |
| Malaria | 29 |
| Stool (worms) | 21 |
| Erythrocyte sedimentation rate | 20 |
| Haemoglobin | 13 |
| Full blood count | 10 |
| Widal (typhoid) | 9 |
| Blood glucose | 7 |
| VDRL (syphilis) | 2 |
| Brucellosis | 1 |
| Creatinine | 1 |
| H. Pylori | 1 |

## For-profit and not-for profit sub group analysis

| Table A10: Effort and quality outcomes from sub-group analysis | | | | |
| --- | --- | --- | --- | --- |
|  | Correct management | | Any unnecessary care | |
|  | For-profit | Not-for-profit | For-profit | Not-for-profit |
|  | Relative risk | Relative risk | Relative risk | Relative risk |
| Base model |  |  |  |  |
| IRT effort | 1.73 (1.20 – 2.49), p=0.003 | 1.98 (1.46 – 2.70), p<0.001 | 0.93 (0.86– 1.01), p=0.073 | 0.90 (0.84– 0.97), p=0.007 |
| Base model + provider characteristics |  |  |  |  |
| IRT effort | 1.84 (1.30 – 2.61), p=0.001 | 1.84 (1.33 – 2.53), p<0.001 | 0.93 (0.86 – 1.01), p =0.083 | 0.91 (0.84 – 0.98), p =0.013 |
| HCW female | 1.19 (0.42 – 3.39) p=0.648 | 1.66 (1.10 – 2.50) p=0.017 | 0.92 (0.76 – 1.11), p=0.387 | 0.92 (0.77 – 1.11), p=0.380 |
| Bonus (vs fixed salary) | 2.72 (1.08 – 6.83), p=0.003 | 1.30 (0.77 – 2.17), p=0.325 | 1.07 (0.91 – 1.25), p=0.434 | 1.18 (0.98 – 1.43), p=0.080 |
| % of clinicians doctors | 1.52 (0.26 – 8.93), p=0.641 | 6.49 (1.49 – 28.26), p=0.013 | 0.89 (0.58 – 1.35) p=0.582 | 0.76 (0.46 – 1.24) p=0.271 |
| Base model + provider characteristics + facility characteristics |  |  |  |  |
| IRT effort | 1.90 (1.29 – 2.79), p=0.001 | 1.89 (1.40 – 2.55), p<0.001 | 0.93 (0.85 – 1.00), p=0.060 | 0.91 (0.84 – 0.99), p =0.023 |
| HCW female | 1.22 (0.38 – 3.91) p=0.741 | 1.61 (1.03 – 2.51) p=0.036 | 0.89 (0.73 – 1.08), p=0.230 | 0.89 (0.74 – 1.08), p=0.237 |
| Bonus (vs fixed salary) | 2.17 (0.70 – 6.74), p=0.179 | 1.39 (0.79 – 2.43), p=0.254 | 1.08 (0.90 – 1.28), p=0.413 | 1.19 (0.96 – 1.47), p=0.111 |
| % of clinicians doctors | 0.75 (0.12 – 4.74), p=0.757 | 2.89 (0.55 – 15.10), p=0.209 | 0.85 (0.53 – 1.36) p=0.487 | 0.78 (0.47 – 1.30) p=0.336 |
| Hospital (vs dispensary) |  | 1.61 (0.78 – 3.32), p=0.201 |  | 0.86 (0.70 – 1.06), p=0.165 |
| Health centre (vs dispensary) | 0.65 (0.20 – 2.12), 0.473 | 1.60 (0.838 – 3.06), p=0.159 | 1.14 (0.92 – 1.41), p=0.236 | 0.97 (0.81 – 1.15), p=0.694 |
| For profit (vs not for profit) |  |  |  |  |
| Peri-urban (vs rural) | 2.29 (0.54 – 9.78), p=0.263 | 1.98 (1.11 – 3.56), p=0.021 | 1.41 (1.06 – 1.88), p=0.018 | 0.99 (0.79 – 1.23), p=0.911 |
| Urban (vs rural) | 1.70 (0.37 – 7.87), p=0.496 | 1.08 (0.56 – 2.09), p=0.813 | 1.22 (0.90 – 1.64), p=0.199 | 0.91 (0.73 – 1.14), p=0.431 |
| Any insurance revenue | 2.09 (0.56 – 7.83), p=0.273 | 1.27 (0.62 – 2.59), p=0.516 | 0.92 (0.77 – 1.09), p=0.319 | 1.17 (0.93 – 1.47), p=0.187 |
| Relative risks are from modified Poisson regression models. Base model includes adjustment for SP fixed effects, SP case and SafeCare intervention arm | | | | |
